# Droplet Microfluidics for Food and Nutrition Applications

**DOI:** 10.3390/mi12080863

**Published:** 2021-07-23

**Authors:** Karin Schroen, Claire Berton-Carabin, Denis Renard, Mélanie Marquis, Adeline Boire, Rémy Cochereau, Chloé Amine, Sébastien Marze

**Affiliations:** 1 Food Process and Engineering Group, Wageningen University and Research, Bornse Weilanden 9, 6708 WG Wageningen, The Netherlands; karin.schroen@wur.nl (K.S.); claire.berton-carabin@inrae.fr (C.B.-C.); 2 INRAE, BIA Biopolymères Interactions Assemblages, F-44316 Nantes, France; denis.renard@inrae.fr (D.R.); adeline.boire@inrae.fr (A.B.); remy.cochereau@inrae.fr (R.C.); amine@capsulae.com (C.A.); 3 INRAE, PANTHER, F-44307 Nantes, France; melanie.marquis@inrae.fr

**Keywords:** emulsions, microgels, microparticles, microcapsules, biopolymers, nutrients, encapsulation, reaction kinetics, interfacial properties

## Abstract

Droplet microfluidics revolutionizes the way experiments and analyses are conducted in many fields of science, based on decades of basic research. Applied sciences are also impacted, opening new perspectives on how we look at complex matter. In particular, food and nutritional sciences still have many research questions unsolved, and conventional laboratory methods are not always suitable to answer them. In this review, we present how microfluidics have been used in these fields to produce and investigate various droplet-based systems, namely simple and double emulsions, microgels, microparticles, and microcapsules with food-grade compositions. We show that droplet microfluidic devices enable unprecedented control over their production and properties, and can be integrated in lab-on-chip platforms for in situ and time-resolved analyses. This approach is illustrated for on-chip measurements of droplet interfacial properties, droplet–droplet coalescence, phase behavior of biopolymer mixtures, and reaction kinetics related to food digestion and nutrient absorption. As a perspective, we present promising developments in the adjacent fields of biochemistry and microbiology, as well as advanced microfluidics–analytical instrument coupling, all of which could be applied to solve research questions at the interface of food and nutritional sciences.

## 1. Introduction 

In the early twenty-first century, the fields of food and nutrition are facing important challenges related to feeding more than 7 billion people both quantitatively and qualitatively. To this end, food design requires many aspects to be accounted for, from the role of food composition and structure to food physicochemical stability and food digestion, in order to optimize nutrient delivery in the human body. Recently, it was recognized that food components and their resulting structures, through their interaction and evolution during storage and digestion, play a major role in nutrient release in the gastrointestinal tract, and are thus important factors determining nutrient absorption and bioavailability [1].

The processes that determine the structure and behavior of foods during production, storage, and ultimately digestion take place at nano- and micrometer scales. Often, these processes are fast and interrelated, which makes investigation of real foods rather challenging. To simplify foods, but still work on their intrinsic multiphase structure, many studies are based on colloidal systems, such as gels and emulsions. However, these systems may still be too complex to fundamentally investigate specific mechanisms at the droplet scale. For this purpose, droplet microfluidics recently emerged as a groundbreaking flexible technique for the food and nutrition research community to specifically design food structures with tailored nutritional effects.

Various droplet microfluidic methods are indeed capable of producing very well-defined droplet-based systems and assessing the physicochemical processes relevant to food. In the present review, we focus on microfluidic techniques that offer the possibility to produce and investigate droplet-based systems in all their various appearances, ranging from monodisperse emulsions to complex gelled systems that can be used for controlled release purposes.

To guide readers unfamiliar with microfluidics, the field of droplet microfluidics and its technical aspects are first introduced. Then, fabrication of various food droplet systems with highly controlled structural features and their potential utilizations are described. The fourth section is devoted to the use of microfluidic devices as platforms for lab-on-chip investigation of physicochemical properties and processes at the droplet scale, such as interfacial tension and interfacial rheology in relation to droplet formation and coalescence stability, phase behavior of droplet-forming biopolymer mixtures, and reaction kinetics in droplets, including nutrient release from lipid droplets under digestive conditions and nutrient transport between cell-mimicking aqueous droplets under absorption conditions. Finally, the last section explores other fields for new promising microfluidic setups that could be used to investigate properties and processes which need to be understood to unlock the high potential of food structure and nutrient delivery control.

## 2. Droplet Microfluidics

**Figure 1:**
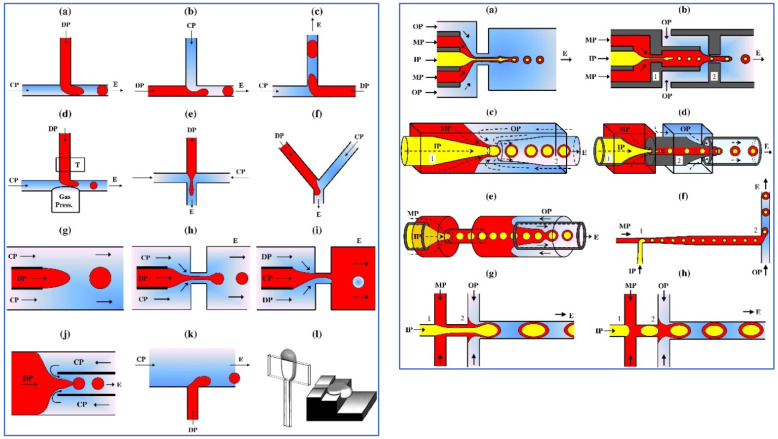
Various microfluidic device geometries and principles. Left panel: simple droplets made in (**a**–**f**) cross-flow junctions with various intersection angles; (**g**) co-flow configuration; (**h**–**j**) flow-focusing configurations; and (**k**,**l**) spontaneous and microchannel droplet formation systems. Right panel: core-shell droplets made in (**a**,**b**) planar co-flow and flow-focusing configurations; (**c**–**e**) capillary co-flow and flow-focusing configurations; and (**f**–**h**) sequential cross-flow junctions. DP, dispersed phase; CP, continuous phase; E, emulsion; OP, outer phase; MP, middle phase; and IP, inner phase. Adapted from [4], with permission from Elsevier.

Microfluidic devices are known for their ability to make very uniform (i.e., monodisperse) droplets [2,3]. This can be achieved using very different microfluidic geometries and principles [4]. Most often, one liquid is dispersed into another using either shear-based methods (e.g., T-junctions or co-flow) or spontaneous droplet formation methods that revolve around Laplace pressure differences (e.g., terrace-based systems). In the following Figure 1 adapted from [4], it can be seen that one liquid flow (or more) can exert a force on another liquid under a certain angle, and that this leads to droplet formation (a–f). In sections (g–j), the two liquids flow in parallel, thus extending the middle liquid, again leading to droplets, that may even contain internal droplets if the design allows for this (right panel). An example of spontaneous droplet formation system is depicted in section (l).

These various methods have been reviewed numerous times, and the reader interested in related mechanisms and products is referred to specialized reviews [4,5,6], and to more recent reviews [7,8,9,10]. Those interested in microfluidic production methods may be interested in [11], and interested in microfluidic tools based on polydimethylsiloxane (PDMS) or even on paper technology [12,13]. A lot of information can also be found on the webpages of producers of microfluidic systems. For those interested in starting with microfluidics, a non-exhaustive list of websites of microfluidic device producers is given in Appendix A.

Although droplet microfluidics has been applied in many different fields ranging from chemistry, in which droplets are used as microreactors, to the medical field, where they can be used as sorting devices, their use in the field of food (even more of nutrition) is still rather limited. Some of the co-authors have written specific reviews within the food field. For shear-based systems, we refer to [14], for spontaneous systems to [15], and more general insights related to the upscaling of microfluidic emulsification methods can be found in [16]. A specific review on lab-on-chip manipulation and study of triglyceride droplets is in [17]. A review on the use of microfluidics in food applications with a focus on the sustainability of food processes is in [18]. The current review is intended to be more comprehensive, and to give more detail on food droplet systems generated and analyzed with microfluidic techniques. Moreover, unlike previous reviews, it considers applications in the nutrition field.

## 3. Microfluidic Fabrication of Food Droplets

### 3.1. Simple Droplets and Emulsions

At the start of microfluidic investigations, most devices were made from PDMS, which is a hydrophobic material, and thus suited to make water-in-oil (W/O) emulsions. In order to make microfluidic devices relevant for food emulsions (most of them being oil-in-water (O/W) emulsions), channel surfaces need to be made hydrophilic, which can be achieved through chemical modification, or by choosing a different construction material (e.g., silicon or glass). Another challenge is to obtain oil droplet sizes in the micrometer scale (1–10 μm), typical of foods. This scale is usually reached with low viscosity alkane oils, but these are inedible oils. To be able to use typical food oils that have a high viscosity and still obtain acceptable oil droplet sizes (in the range 10–100 μm), the geometry and dimensions of the devices need to be considered in detail, in relation to the process conditions used, an important parameter being the flow rate of the liquids [19].

**Figure 2:**
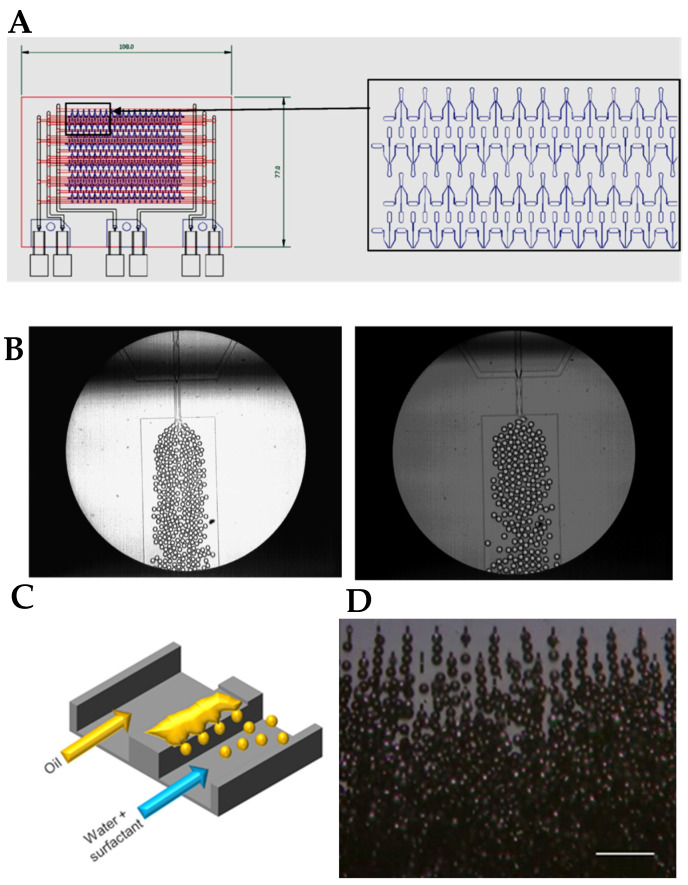
(**A**) Scheme of a PDMS microfluidic platform with parallelization of 20 micro-units for the production of W/O emulsions. (**B**) Droplet generation in two flow rate conditions in a single unit of the microfluidic platform. Adapted with permission from [25]. Copyright (2009) American Chemical Society. (**C**) Schematic representation of EDGE emulsification (reprinted from [29], under the terms of the Creative Commons Attribution 4.0 International license). (**D**) Grooved microfluidic system, picture courtesy of Dr. Kobayashi, Food Research Institute, University of Tsukuba, Japan.

Despite all the limitations, droplet microfluidics could be applied to food emulsion production, with the challenge to place microfluidic devices in parallel to reach throughputs higher than the current limit of about 1 L h^−1^ [16,20]. For example, in co-flow devices, as reported in [21], a challenge is to connect all channels in such a way that they are all operational, mostly making the actual chip a tiny part of a web of connected tubes. Very early on in 2002, in the group of Nakashima, grooved microfluidic systems were developed that have a general feeding area that connects to all droplet formation units, producing extremely uniform droplets at high throughput [22] (Figure 2D). Note that these straight-through systems were also used in later studies to make double emulsions [23]. In later work, various viscosity ratios were investigated, leading to scaling relations for emulsions relevant for food applications [24].

Another pioneering work was performed at Unilever Colworth, where a scaled-up microfluidic system was used for the production of monodisperse water-in-sunflower oil emulsions [25]. This system did not require active control on each of the 20 production units within the microfluidic platform, and yielded emulsions with a narrow droplet size distribution centered around 20 μm (Figure 2A,B). Other approaches to scale-up were reported, such as a three-dimensional monolithic elastomer device for mass production of monodisperse emulsions [26]. Using double-sided imprinting, 3D microchannels were formed in a single elastomer piece that had 1000 parallel flow focusing generators. With this approach, W/O emulsions could be produced at high throughput, with a 45 µm droplet size. Comparison to other parallelized systems in terms of droplet size and throughput can be found in [26,27].

Besides parallelizing microfluidic units, many droplets can also be produced from one droplet generation unit. In EDGE systems (Figure 2C), and later in STEP systems, multiple droplets can indeed be formed simultaneously [28,29]. The system is based on a shallow area over which an oil film flows, and then breaks up into many droplets upon reaching a deeper area. Although some important improvements have been made in the design of these devices, approaching the micrometer scale with vegetable oils [30], the throughput is still such that a full-scale food product is not within reach. Production of food additives, however, is within reach given current production rates, especially through novel techniques such as in-air microfluidics [31], which can be used to efficiently encapsulate oil droplets in the 1–500 μm range.

### 3.2. Multiple Droplets and Emulsions

As for simple emulsions, many microfluidic methods exist for the production of multiple emulsions, usually based on a one-step (using capillaries) or a two-step (using microchannels) droplet generation. They are frequently water-in-oil-in-water (W/O/W) or oil-in-water-in-oil (O/W/O) double emulsions, but can also comprise more intermediate phases (for triple, quadruple, and quintuple emulsions, see [32]). Their microfluidic production was the subject of both basic and applied research, with pharmaceutical, food, or cosmetic applications [6]. Notably, microfluidic-made double emulsions present the advantages of being monodisperse and that the number of their internal droplets is well controlled. These features make them very promising to overcome the usual poor physical stability of double emulsions produced conventionally.

**Figure 3:**
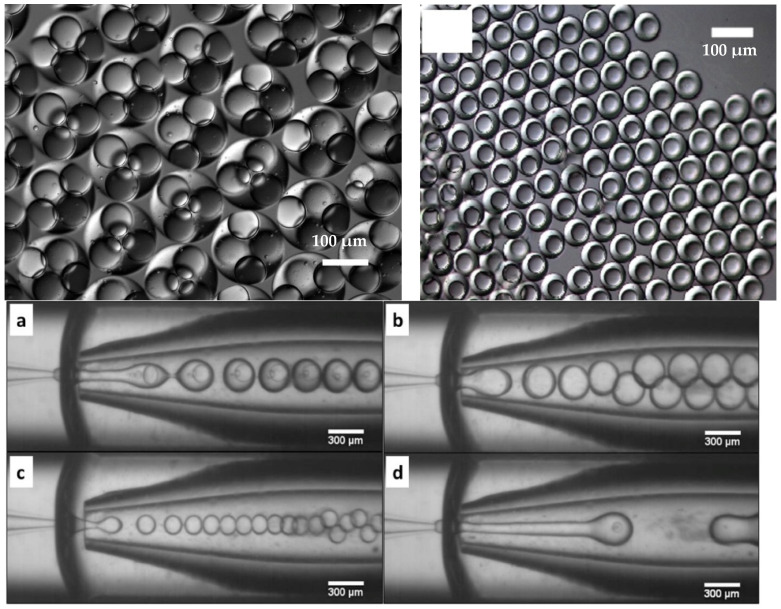
Examples of monodisperse W/O/W emulsions produced in a glass microchannel device (top) or in a glass capillary device (bottom, **a**–**d**). Reprinted from [33] and [36], respectively, with permission from Elsevier.

As mentioned earlier, the straight-through microchannel device was used for the first production of W/O/W emulsions made of food grade components [23], although this first attempt resulted in poorly controlled internal water droplets. Another microchannel production of W/O/W emulsions was achieved using a glass device that allowed control over the number of internal water droplets and the external oil droplet size, as shown in Figure 3 [33]. The authors connected a hydrophobic device and a hydrophilic device and, through variation of process conditions, were able to vary the number of droplets. Such high control of the structural features of the W/O/W emulsions enabled the internal droplet size change to be monitored by NMR.

The more widespread glass capillary technology was used to produce W/O/W emulsions encapsulating water-soluble food pigments. Color retention was found to remain high for at least 30 days, especially at low temperature and low pH [34]. A similar glass capillary device was used to produce W/O/W emulsions with an evaporable oil phase containing phospholipids and beta-carotene in order to form giant liposomes encapsulating beta-carotene in their membrane. These liposomes were physically stable for 7 days at room temperature [35]. Another glass capillary device was used to produce W/O/W emulsions encapsulating sucrose. Emulsions with multiple internal water droplets were not stable upon storage, whereas those with a single internal water droplet were stable for at least 60 days. This resulted in high or low sucrose release upon in vitro oral digestion, respectively, increasing with decreasing oil-soluble internal emulsifier (PGPR) concentration [36].

Recently, a PDMS microchannel device was used to produce W/O/W emulsions with a single internal water droplet encapsulating *E. coli*. The bacteria release was found to depend on salt concentrations in both water phases, and also on the water-soluble external emulsifier (Tween 80) concentration [37].

### 3.3. Microgels and Microparticles

Microparticles able to transport and release bioactive compounds are being increasingly used for food applications, as they provide a variety of functionalities. To date, various strategies for the production of polymer microgels exist. However, most of these methods involve multistep processes, do not use biocompatible components, or do not allow for precise control over the dimensions and internal structure of the microgels [38]. In this context, natural polymers present wide advantages, due to their biocompatibility and biodegradability, for the elaboration of bio-based polymer microgels in the size range from several micrometer to hundreds of micrometers, as used in the pharmaceutical, cosmetics, nutrition, pesticide, and food industries [39,40]. Alginate is one of the most used natural polymers due to its biocompatibility and its ability to form a three-dimensional network, called hydrogel, in the presence of divalent cations [41].

**Figure 4:**
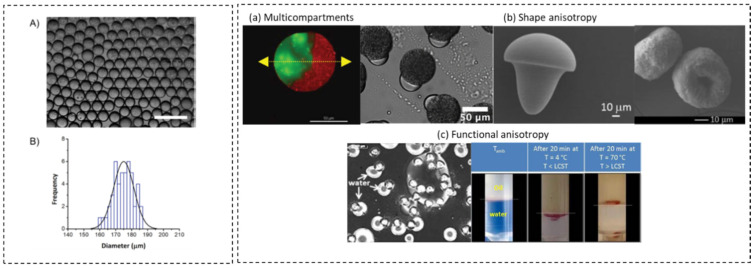
Control of the shape, size, and composition of microparticles and microcapsules produced by droplet microfluidics. (**A**,**B**) Size distribution of agarose-gelatin microgels obtained by the control of the flow rates in a flow-focusing device, adapted with permission from [43]. Copyright (2014) American Chemical Society. (**a**) Multicompartment and (**b**) anisotropic microparticles with Janus, mushroom, or doughnut morphologies and amphiphilic properties, adapted with permission from [45,46,49]. Copyright (2012) (2014) (2020) American Chemical Society. (**c**) Functional anisotropy as an amphiphilic property, reprinted from [47], with permission from Elsevier.

In the last two decades, microfluidic strategies for the production of natural polymer particles have become available, featuring the use of food-grade biopolymers and precise control over the morphology and size of biopolymer colloids, size ranging from 60 µm to 175 µm (Figure 4A) [42,43,44].

Multicompartment and anisotropic particles have received significant attention due to their novel morphologies and diverse potential applications. Droplet microfluidic techniques offer possibilities to control the morphology and spatial composition of these particles, as shown in Figure 4B sections (a–c). For example, the formation of Janus particles (i.e., particles with two hemispheres of distinct properties) using two chemically distinct biopolymers and ionically cross-linked hydrogels was achieved. A microfluidic device for the generation of monodisperse homo and hetero microbeads using pectin−pectin and pectin−alginate hydrogels was developed [45]. Additionally, pectin microparticles were produced using droplet microfluidics with dimethyl carbonate as an organic continuous phase. Sphere, doughnut, oblate ellipsoid, or mushroom morphologies were obtained, demonstrating the ability to control the formation of anisotropic biopolymer-based hydrogel microparticles [46]. Functional anisotropy was later demonstrated by the production of monodisperse responsive alginate-block-polyetheramine copolymer microgels presenting amphiphilic properties [47]. This droplet microfluidic technique was also adapted to generate bio-based and responsive Janus microparticles, used as new colloidal stabilizers for dispersed systems such as Pickering emulsions [48]. Other studies dealt with the scalable synthesis of Janus particles using eco-friendly polymers and processes [49].

Microparticle morphology may also control the release of encapsulated compounds. For example, a model protein, bovine serum albumin (BSA), was encapsulated within marine exopolysaccharide microparticles in a one-step process using droplet microfluidics. Microparticle morphology was controlled by polysaccharide molecular weight and concentration. Protein release was tuned by the microparticle morphology, with lower release from the more homogeneous structures [50]. A recent article reported the efficient generation of bio-based hybrid microparticles using droplet microfluidics. This hybrid protein/polysaccharide microparticles offered advantages due to protein–protein and protein–polysaccharide interactions. As a proof of concept, egg white/carrageenan microparticles loaded with a hydrophilic compound (dye yellow sunset) were produced, their transport and release properties depending on the cross-linking method [51].

### 3.4. Microcapsules with Single and Multiple Oil Cores

**Figure 5:**
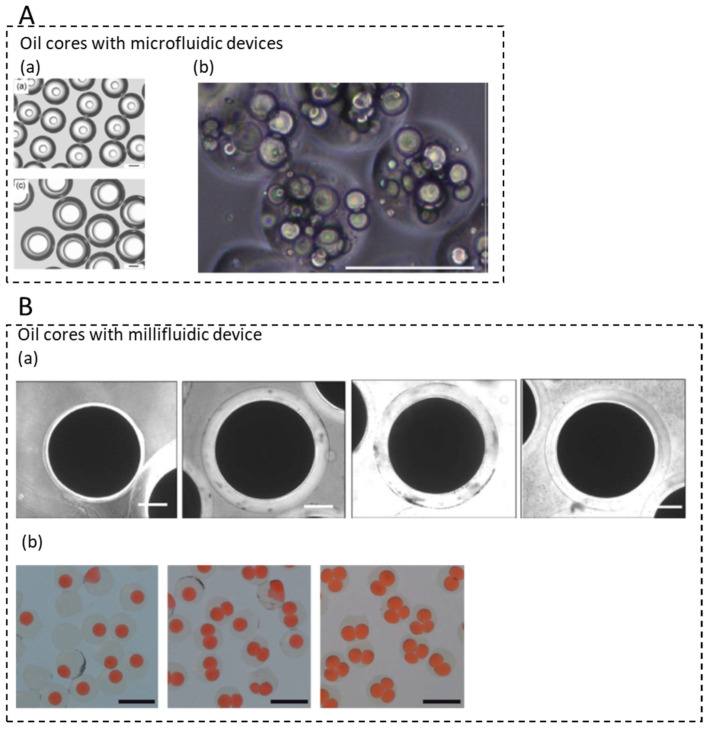
Oil core/aqueous shell microcapsules. (**A**) Microcapsules by droplet microfluidics: (**a**) Control of the alginate membrane thickness (reprinted from [53], with permission from Elsevier), and (**b**) multiple oil cores by a two-step approach using Pickering emulsion (adapted with permission from [55]. Copyright (2016) American Chemical Society. (**B**) Capsules by droplet millifluidics: (**a**) Control of the alginate membrane thickness of capsules with a diameter of 1 mm and an oil core (reprinted from [57], with permission from Elsevier), and (**b**) control of the number of oil cores in alginate capsules (reproduced from [59], with permission from The Royal Society of Chemistry).

Capsules can be classified according to their morphology, size, and the number of encapsulated cores. Capsules have found applications in several areas, including food, and they can be produced by several techniques: jet-cutting, vibrating jet, spray-drying, dispersion, and milli/microfluidics [52]. The advantage of milli/microfluidic systems consists in the formation of monodisperse capsules with precise control over their size (and membrane thickness) and composition, including oil loading as shown in Figure 5 [53,54].

Oil encapsulation using alginate as encapsulating material can be carried out using technologies based on an external, internal, or inverse gelation mechanism, coupled with droplet microfluidics [44]. Recently, the production of alginate microgels containing multiple oil cores was reported using a two-step method involving a Pickering emulsion in a droplet microfluidic device. Lipophilic compounds were encapsulated within Pickering emulsion droplets covered by alginate microgels, which resulted in better protection and sustained release profile compared to capsules made by conventional methods (Figure 5A section (b)) [55]. A capillary microfluidic device was recently described for the generation of spherical and bullet-like alginate microcapsules with a core-shell structure, based on the deformation of double emulsion droplets. The release performance of these microcapsules was investigated in detail by encapsulating α-tocopherol. The results show that the bullet-like microcapsules exhibit faster release compared to the spherical microcapsules [56].

At another scale, droplet millifluidic devices can produce monodisperse emulsions that may be used as template to core-shell capsules for food applications. For example, in Figure 5B section (a), an inverse gelation method to produce alginate (micro)-capsules with a narrow size distribution using droplet millifluidics was developed. A W/O emulsion with a dispersed phase containing Ca^2+^ ions was directly injected into a continuous alginate phase to generate a secondary W/O/W emulsion. Due to the cross-linking of alginate molecules by Ca^2+^ ions release, core-shell (micro)-capsules were formed with a very high oil loading. Monodisperse core-shell capsules with sizes ranging from 140 µm to 1.4 mm were produced by tuning flow rates of the continuous and dispersed phases and by varying the internal diameter of the capillary tubing [57]. Compression experiments on individual capsules were later performed and revealed that their mechanical properties were inversely related to alginate membrane thickness, i.e., the thicker the membrane, the lower the surface Young modulus. This result was explained in terms of enhanced swelling properties of the alginate membrane with curing time or specific storage conditions. Dried capsules had even more resistant membranes due to the loss of water [58].

At the frontier of droplet millifluidic and dropping methods, a millifluidic pendant drop method, allowing the production of double emulsions in a controllable way, was developed (Figure 5B section (b)). Using a co-flowing drop-maker, a periodic train made of monodisperse droplets was generated and directed toward the end of a capillary tube to form a pendant drop. When this drop detached from the tip of the capillary under the influence of gravity, it could encapsulate one or several droplets, depending on the experimental conditions. Well-calibrated, large emulsion gels were thus produced with alginate beads filled with droplets, the number of droplets being controlled by the ratio of the production times [59].

Alternatively, systems that use in-air microfluidics have been described for the production of oil-filled microgels. For example, emulsions were mixed with an alginate solution and subsequently extruded from a syringe under air flow, leading to very well-defined microgels. The release of these gels could be controlled with the mesh size of the gel and the size of the beads. This technique could be scaled up and used to put multiple droplets in microgels [31].

### 3.5. Microcapsules from Water-in-Water Emulsions

**Figure 6:**
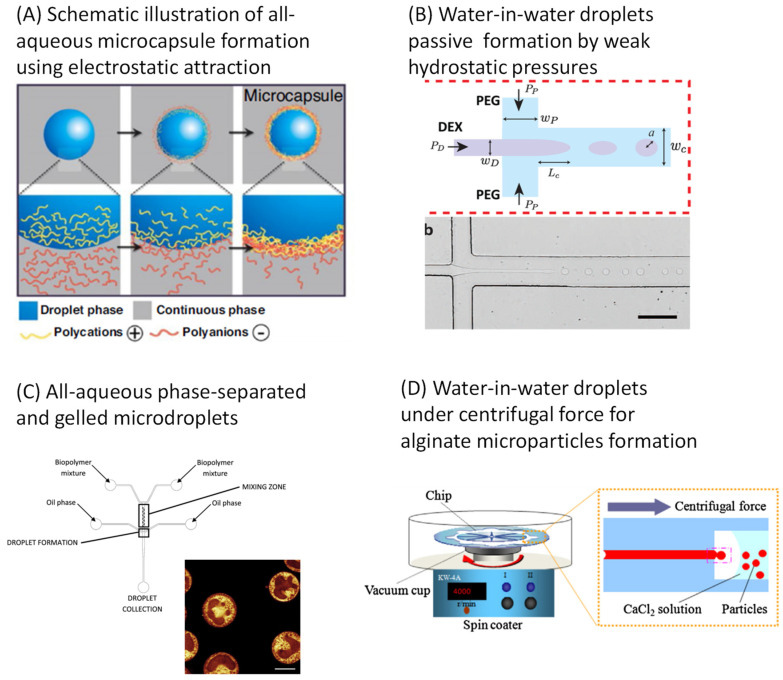
Schematic principles of all-aqueous microcapsule formation. (**A**) Reprinted from [60], under the terms of the Creative Commons Attribution 4.0 International license. (**B**) Adapted with permission from [66]. Copyright (2016) American Chemical Society. (**C**) Reprinted from [63], with permission from Elsevier. (**D**) Reprinted from [65], with permission from Elsevier.

All-aqueous emulsions are conventionally formed at bulk scale by mild shaking of aqueous two-phase systems (Figure 6A) [60]. Although interfacial tension, which generally plays a major role in microfluidic emulsification, is very low in such systems, droplet microfluidic methods could be developed for the compartmentalization of aqueous polymer solutions within water-in-water microdroplets, in the absence of organic solvent and surfactant [61]. Phase separation inside droplets was also used to produce all-aqueous core-shell structures using droplet microfluidics [62]. Other authors produced biopolymer particles using the phase separation and gelation properties of gelatin-maltodextrin mixtures in a microfluidic device involving flow-focusing and passive mixing (Figure 6B). By controlling the production of biopolymer droplets as well as their gelation and phase separation kinetics, the microstructures inside the gelled particles were found to be highly reproducible [63].

Some authors investigated the use of microchannel emulsification to produce monodisperse gelatin/acacia complex coacervate microcapsules of soybean oil. A surfactant-free system was developed. First, microchannel emulsification using gelatin was compared with that using decaglycerol monolaurate. A high concentration of gelatin was found to promote the production of regularly sized droplets, and surfactant-free monodisperse oil droplets produced by microchannel emulsification could be microencapsulated within the coacervate [64]. In another work, a novel approach was proposed for the fabrication of alginate microparticles by using a circular chip composed of multiple radial channels. Here, water-in-water emulsions were generated under centrifugal force, and the gelation occurred directly in the chip. The microfluidic chip was fabricated by soft lithography using PDMS, and microparticle production rate could be increased through the number of channels (Figure 6C). Moreover, the chip was suitable for the generation of alginate Janus microparticles [65]. A microfluidic system that generates water-in-water droplets by passive flow focusing was also proposed (Figure 6D). Droplet formation was achieved by applying weak hydrostatic pressures to generate low-speed flows at the flow focusing junction. Although this system was able to produce food-grade microcapsules of 10–40 µm, the production rate was still rather low, in spite of reported up-scaling options [66].

These all-aqueous droplets are expected to find many food applications, as their compositions are biocompatible, and their structures can be finely tuned, as recently shown in [67], where double and even triple all-aqueous emulsions based on PEG and dextran were produced using a hybrid PDMS/glass capillary microfluidic device. However, one of the main concerns related to all-aqueous systems produced by microfluidics is the ease of droplet formation in the absence of an appreciable interfacial tension, which implies that shear forces need to be tailored precisely. This is possible, but could become very challenging in up-scaled devices. For systems relying on polymer interactions, it is also important to minimize interactions with the construction material(s) of the microfluidic chip, which may have a large influence on the production.

## 4. On-Chip Analysis of Food Droplets

### 4.1. Interfacial Properties and Droplet Stability

A specific aspect related to food that is often missing in basic microfluidic science is the presence of surface-active components. On the one hand, this facilitates droplet formation in microfluidic devices, as surfactants adsorb to the oil-water interface. On the other hand, this may complicate droplet formation due to interactions with the construction material(s), leading to wettability changes that are generally undesirable [29,68]. In the section below, we highlight how microfluidic devices can be used to monitor fast processes at small length scales, such as adsorption or coalescence taking place during and just after droplet formation.

#### 4.1.1. Interfacial Tension

**Figure 7:**
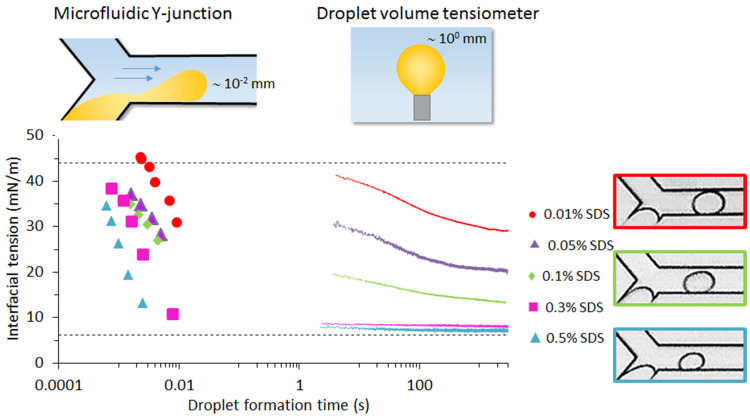
Schematic representation of a microfluidic Y-junction (top left) and of a droplet volume tensiometer (top right). The bottom graph represents the interfacial tension at the hexadecane-water interface with various SDS concentrations in the aqueous phase, as measured with a microfluidic Y-junction (symbols) and with a droplet volume tensiometer (solid lines). The dotted lines represent the equilibrium interfacial tensions without (top) and with surfactant (bottom). Images on the right show droplets made with increasing SDS concentrations. Adapted from [72], with permission from Elsevier.

In general, the size of droplets created in a microfluidic device can be predicted using a force balance that links the shear force and the interfacial tension in the liquid neck that keeps the droplet connected to the feed channel, as would be the case in classic T-junctions [69]. Mostly, a capillary number (*Ca*) is used for this, as was demonstrated in [70] for Y-junctions operated within an extreme working range. While interfacial tension is kept constant in many fundamental studies, this is not the case in food-related systems, but the logic described above can be reversed. Thus, from a measurement of the droplet size, the interfacial tension at droplet formation can be derived, if the mechanism of droplet formation in the absence of surface-active components is known precisely [71]. The droplet size obtained for various flow conditions and surfactant concentrations (see pictures in Figure 7) can thus be used to calculate the interfacial tension using a calibration curve obtained in the absence of surfactant [72].

In first attempts to do so with sodium dodecyl sulfate (SDS) and Tween 20, T-junctions were used in both experimental and modelling studies [73]. In follow-up works [72,74,75,76], it was shown that effects due to surfactants adsorbing from the continuous phase, from the dispersed phase, and the convective mass transfer could be distinguished. The time within which droplet formation was studied (and thus interfacial tension kinetics during droplet formation) was in the (sub)millisecond range, allowing observations within a time scale that occurs in industrial emulsification devices [77]. In Figure 7, data obtained using a droplet tensiometer, classically used for interfacial tension measurements, are shown for comparison, clearly showing the orders of magnitude difference in time ranges. The graph shows that the acting interfacial tension would be underestimated at very short droplet formation times if equilibrium values were used.

Some work was also undertaken on protein adsorption, and the results were compiled into dimensionless numbers. The interfacial tension played an important role, together with the applied shear force, and the expansion rates during premix membrane emulsification, and very reasonable predictions of the droplet size were obtained. These results show that translation of microfluidic observations to large-scale processes is possible to some degree [68,78].

#### 4.1.2. Coalescence Stability

**Figure 8:**
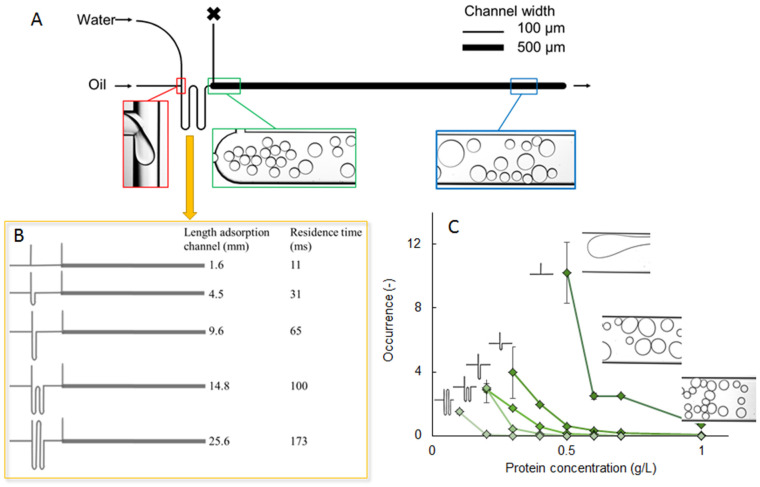
(**A**) General design of the coalescence chip. The droplets are generated at the T-junction (red square), after which they are transported through the meandering channel (which may have different length—panel **B**) to the coalescence chamber where images are collected (green and blue squares in panel **A**), and related to the number of coalescence events that took place (reprinted from [83], with permission from Elsevier). (**C**) Typical data from the coalescence chip, showing the occurrence of oil droplet coalescence for various concentrations of pea proteins in the aqueous phase, when systematically changing the stabilization time through the length of the meandering channel. The microscopy pictures on the right of panel **C** illustrate typical appearance of the droplets at the exit of the coalescence chamber [84], under the CC BY license 4.0.

Interfacial tension is linked to surface coverage by surface-active molecules, and thus related to droplet coalescence. Pioneering work using microfluidic tools to investigate surfactant-covered droplets was undertaken by Krebs et al. [79,80,81]. Experimental setups are usually based on one or more droplet generation units producing droplets that interact with one another in an observation area (Figure 8, top panel). Most studies have revolved around surfactant behavior (e.g., [82]), whereas only very limited information is available on protein behavior [83,84].

The coalescence chips are designed in such a way that decouples droplet formation and interaction between droplets, which is not possible in classic emulsification devices. The time allowed for adsorption to take place can be varied through the length of the meandering channel (and the applied flow rates; see Figure 8B). Droplets are next allowed to interact in a wider coalescence chamber, and their size is monitored at the entrance and exit of the chamber. The number of coalescence events they underwent can be calculated straightforwardly, as the droplets are very uniform in size at the T-junction creation point. When plotting the coalescence occurrence as a function of protein concentration (Figure 8C) for different meandering channel lengths, it is possible to compare various process conditions, and how they affect emulsion stability. The translation from the microfluidic observations to larger-scale effects is still an active research field, but it is clear that the insights obtained by microfluidic observations are instrumental in comparing the effectiveness of various surface-active components to stabilize droplets. In addition, this method has shown to be quite versatile in the tested applications, and allows us to pinpoint effects that are often not considered in emulsion formulation, such as the effect of protein oxidation [84], or the destabilizing versus stabilizing roles of colloidal particles in Pickering emulsions [85]. The coalescence chip has also been used at elevated temperature, as described in Section 4.1.4.

#### 4.1.3. Interfacial Rheology

**Figure 9:**
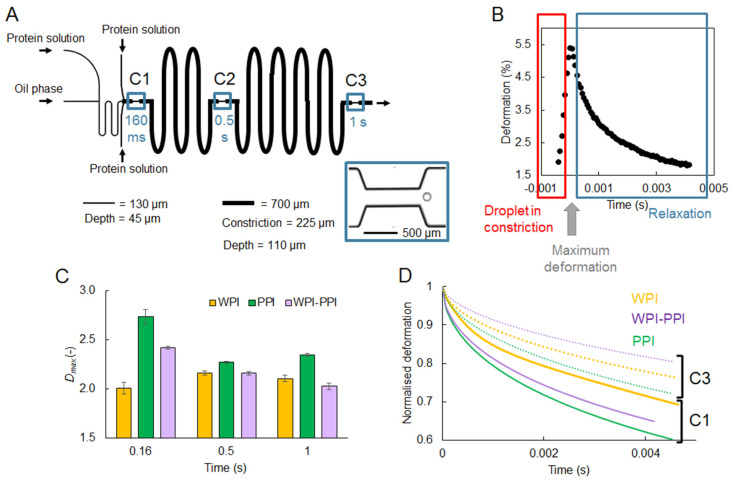
(**A**) Layout of the microfluidic rheology chip. The T-junction where droplets are formed (on the left) is the same as in the coalescence chip (Figure 7). The three constrictions (C1, C2, and C3) have a 70° angle. (**B**) Typical deformation pattern in time recorded by image analysis through passage of an emulsion droplet in a constriction. (**C**) Examples of maximum droplet deformations measured for droplets stabilized by different proteins (WPI, whey protein isolate; PPI, pea protein isolate; WPI-PPI, blend of both in 1:1 mass ratio), at the different constrictions C1, C2, and C3. (**D**) Examples of normalized relaxation curves for droplets stabilized by the same proteins, at constriction C1 (solid lines) or C3 (dotted lines). Reproduced from [89], under the CC BY license 4.0.

Besides the interfacial tension as such, which is related to the ease of droplet formation and to the propensity of droplets to coalesce, the rheological behavior of the interfacial layer is another aspect that also contributes to emulsion stability, and can be investigated using microfluidic techniques. For protein-stabilized emulsions, it is generally assumed that the formation of the interfacial layer structure takes place in time ranges between minutes and days. In previous studies, authors investigated interfacial rheology using microfluidics at time intervals up to hours, and found considerable differences in interfacial rheology [86,87,88]. However, a very recent study showed that the onset of these processes can already be detected at sub-second time scales [89]. In this work, protein-stabilized droplets were subjected to successive deformations by flowing through a series of constrictions in a so-called rheology microfluidic chip (Figure 9A). The deformation of the droplet and relaxation thereafter was investigated through image analysis (Figure 9B), and it was found that at time scales well below 1 s, the onset of film formation could be detected. Furthermore, various proteins (alone or in mixtures) were used to stabilize droplets, and insights in the mechanical strength of the interfacial layer surrounding the droplets could be obtained (Figure 9C,D). For instance, interfacial films made of whey proteins (WPI) showed a much higher resistance against deformation compared to films made of pea proteins (PPI), or of mixtures of both proteins (Figure 9C). A series of constrictions in the chip design also allowed for probing an increased viscous contribution in the interfacial rheological properties over the relevant time span (typically, between the first and last constrictions, C1 and C3, respectively; Figure 9D). The insights obtained with such tailor-made microfluidic devices help to capture effects at short time scales, and are thus relevant to unravel phenomena occurring in industrial processes.

Studying droplet deformation at the millisecond scale in a microfluidic device was also applied to assess the degradation of frying oil. By kinetic measurements of the deformation of water droplets in frying oil after their flow-focusing generation, the time at maximal deformation, as well as the final water droplet diameter, were found to be correlated to the frying oil total polar materials, a degradation indicator. This degradation was also found to increase oil viscosity and decrease equilibrium interfacial tension [90].

#### 4.1.4. Temperature and Pressure Sensitivity

**Figure 10:**
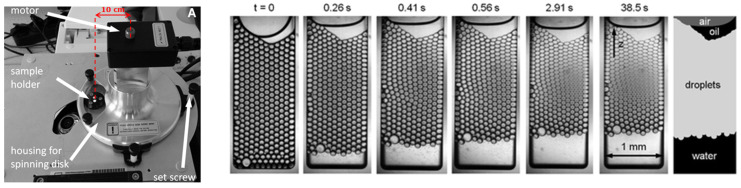
Left: Picture of the experimental setup with the microfluidic chip on the centrifuge. Right: Series of images recorded during a compression experiment with a relative g-force a/g = 231, with a the radial acceleration and g = 9.81 m s^−1^ the normal gravitational acceleration. The enhanced gravity force acts along the z-direction. The emulsion is a hexadecane-in-water emulsion stabilized by 10 mM SDS. The droplet diameter is 97 μm. Upon centrifugation, the droplet layer is compressed, and a layer of pure aqueous phase forms at the bottom of the sample chamber, as displayed in the scheme on the right. After 2.91 s, a steady-state height of the droplet column is reached (reproduced from [91], with permission from The Royal Society of Chemistry).

Very limited microfluidic work has been dedicated to probing extreme conditions. Thus, it is useful to share the latest methods available to probe such aspects, and show their potential relevance for food studies. In principle, glass chips are resistant to temperature and pressure, and this has been investigated in a pioneering work using a small microfluidic container in combination with a centrifuge mounted onto a microscope connected to a high-speed camera [91]. The authors were able to monitor how accelerated gravity conditions influence droplet deformation and coalescence (Figure 10).

The same set-up has also been used to investigate the effect of a temperature-sensitive surfactant on emulsion stabilization when exposed to different temperature triggers [92]. In doing so, it was possible to measure fundamental properties of this system, such as the critical disjoining pressure, as a function of temperature. Although these measurements were carried out in surfactant-based emulsions, it is clear that, given the process conditions to which foods are exposed, these methods are also relevant for the food field.

In a recent work [93], the coalescence cell was used at temperatures up to 70 °C, and coalescence of emulsion droplets stabilized by a food-grade surfactant (Tween 20) was compared with that of droplets stabilized by a model non-food surfactant (SDS). Both surfactants showed similar behavior, with coalescence frequency increasing with temperature. This was related to a decrease in viscosity at higher temperatures, which triggers a stronger perturbation in the aqueous thin film separating droplets, leading to more film rupture. Work is on-going on protein stabilized droplet coalescence, making it even more relevant for food applications.

### 4.2. Screening of Biopolymer Phase Behavior Using Microfluidic Technologies

The solubility of biopolymers is a central issue in the development of new applications in food science and technology. This solubility depends on the physicochemical conditions of the solvent (pH, ionic strength, temperature, etc.), and can be modulated by the addition of ions or macromolecules. A way to probe the solubility and the assembly state of a biopolymer is to build its phase diagram [94,95]. A phase diagram maps the state of the dispersion (liquid, coacervate, gel, crystal, or glass) as a function of the biopolymer concentration and the quality of the solvent. This implies screening a wide range of physical chemical conditions. This is usually performed in tubes, but this is time-consuming and requires large amounts of raw materials, which are not always available. Alternative strategies based on microfluidics have been developed in the past 15 years that improve the screening efficiency. Such strategies will be reviewed in the following section, from the simplest to the most advanced technology.

One of the first millifluidic-like devices, designed to study polymer phase diagrams as a function of temperature, was based on glass capillaries [96]. Rectangular capillaries of a few microliters, with a cross-section of 0.1 × 1 mm^2^, were placed perpendicularly to two channels containing, respectively a hot and a cold fluid, providing a temperature gradient along the capillaries. The onset of phase separation was detected by optical microscopy in dark field mode. Measurements were taken at several polymer concentrations [96] and ionic strengths [97]. However, the fact that each tube had to be filled individually limited the screening ability.

Significant improvements were made by using continuous flow and mixing devices. A simple droplet-based millifluidic device was developed to study the crystallization of mineral, organic, or biological compounds, based on the assembly of 0.5 mm Teflon capillaries. Droplets were generated in a continuous inert phase, reducing the amount of raw material needed [98,99,100]. The size and composition of the droplets were modulated by varying the flow rates. An on-line UV detector was used to determine the concentration in each droplet [100]. Droplets were then stored and incubated in a thermostatic bath to study the impact of temperature on crystallization. This simple system has a low implementation cost, is compatible with many solvents, and allows the circuit to be modulated according to needs.

**Figure 11:**
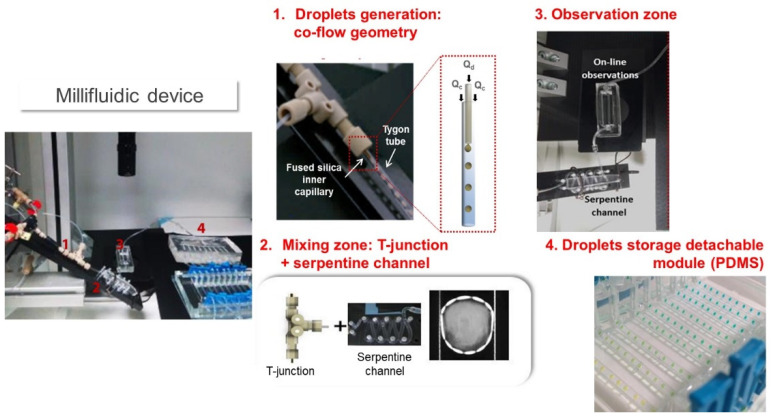
Droplet millifluidic devices developed to build biopolymers phase diagrams (adapted from [102] with permission from Springer).

Later, a similar system (Figure 11) was developed to study biopolymer mixtures [101,102,103]. The main challenge was to provide an efficient mixture of viscous dispersions, enhanced by mixing T-junction-like geometries and winding channels. Placed after droplet formation, these winding channels were very efficient in promoting chaotic advection and, therefore, mixing [102,103,104,105]. Such a channel shape was suggested to induce liquid stretches and folds within the droplets, similar to the baker’s transformation [106]. The use of droplet-based millifluidics to screen biopolymer mixtures was found to reduce the amount of material and the experimentation time by a factor of ten and five, respectively, compared to a conventional bulk approach [102]. One of the limitations of such a droplet-based system is the presence of an oil-water interface where biopolymers, particularly proteins, could adsorb. Thus, it cannot be used in very diluted biopolymer dispersions for which the percentage of adsorbed biopolymer is not negligible compared to the initial bulk concentration [101].

One important advantage of microfluidic chips based on photolithography is the possibility of integration of various functions on a single chip. It may indeed be designed to successively formulate droplets, generate droplets, mix droplets, tune solute concentration through water permeation, and store droplets under controlled osmotic conditions [107,108]. The pervaporation of the water through PDMS membrane also provides an effective way of tuning the solute/biopolymer concentrations [109,110,111]. Parallelized channel arrays increase the screening ability as compared to millifluidic systems. In some cases, PDMS is replaced by Teflon, making it possible to study phase transitions in non-aqueous solvents [112]. PDMS can also be combined with glass capillaries to allow droplet collection for storage or later analysis [113]. Such microfluidic platforms were, for example, used to probe polymer [108,114] and protein phase diagrams [107,108,113]. This is of particular interest for the investigation of protein crystallization [107,108,115]. It allows for the growth of high-quality crystals, free from convective instabilities due to turbulence, as reviewed in detail in [115]. The precise control over mass and heat transfers also provides a good control over kinetic paths [115].

Recently, several droplet-based microfluidic devices were developed to produce size-controlled aqueous core-shell particles such as liposomes, polymersomes, or proteinosomes [116,117,118,119,120,121]. These structures allow for molecule exchanges through their permeable interface between their closed aqueous core and an external aqueous phase. This offers an innovative way to change the physicochemical conditions of a solution, and therefore to study the phase behavior of biopolymers within a microenvironment. In the case of liposomes, the exchange of small (<2 kDa) molecules through the shell, composed of a lipid bilayer, can be induced by the formation of nano-pores [118,122,123,124,125,126]. Based on the use of liposomes and nano-pores, microfluidic techniques were developed to tune and study the phase behavior of biopolymers [127,128,129]. In the latter study, a single chip allowing the production and trapping of giant unilamellar vesicles encapsulating biopolymers was developed. Theses vesicles were produced from W/O/W double emulsions in less than 30 min after detachment and solubilization of the oil phase. The vesicles were transformed into semi-permeable vesicles, also called osmosomes, after pore formation. The chip was designed to tune the buffer composition of the osmosome in less than one minute. This provided a way to control the kinetics of buffer exchange and to probe the first stages of biopolymer assembly. A proof of concept for biopolymer assembly within osmosomes was established by pH-triggered liquid–liquid phase separation of wheat proteins within a few minutes [129].

### 4.3. On-Chip Monitoring of Reaction Kinetics

A recent promising food and nutrition application of droplet microfluidics is the on-chip quantification of various reaction kinetics linked to food or biological droplets and their bioactive contents, with high control of structural features. This approach is recent compared to the characterization of the reaction and release properties of droplet-based microparticles generated on-chip but analyzed off-chip, as described in Section 3.

The seminal work of Huebner et al. [130] demonstrated the potential of microfluidic chips including individual droplet trapping for the investigation of reaction kinetics of several biological processes (aqueous droplet solubilization, aggregation of *E. coli* cells, and a β-galactosidase enzymatic reaction). A similar microfluidic strategy was also used to trap giant unilamellar vesicles and study the release kinetics of small molecules (calcein or tagged dextran) from their aqueous core, which was found to depend on protein-induced pore formation and lipid membrane composition and structure [124,131]. As described earlier, a similar approach was also used to modify the buffer composition and pH in the aqueous core of vesicles. The kinetics of buffer exchange probed by calcein release could be finely tuned by the concentration and flow rate of the pore-forming protein solution [129].

The application of this droplet trapping approach to food and nutrition was initiated by Marze et al. [132] for on-chip monodisperse oil droplet formation, individual trapping, and gastrointestinal digestion kinetics monitoring. Using various types of triglyceride as the oil phase, they corroborated classical findings usually obtained through in vitro digestion of polydisperse emulsions. Notably, they confirmed that the type of triglyceride controls the digestion kinetics, however at a fatty acid release rate per unit surface area, about 10-fold faster than for the corresponding emulsions. This result was attributed to the absence of flocculation or coalescence between droplets in the microfluidic method, which typically induces a slowdown of the digestion kinetics in the emulsion method.

The effect of oil droplet coalescence on digestion kinetics was studied using a similar microfluidic device, where several droplets could be confined in the same trap [133]. Their digestion kinetics was indeed found to be slower compared to that for a single droplet trapped individually. This effect was attributed to the reduced specific surface area for coalesced droplets, confirmed by showing that the digestion rate of individually trapped droplets was lower as droplet size increased (specific surface area decreased).

**Figure 12:**
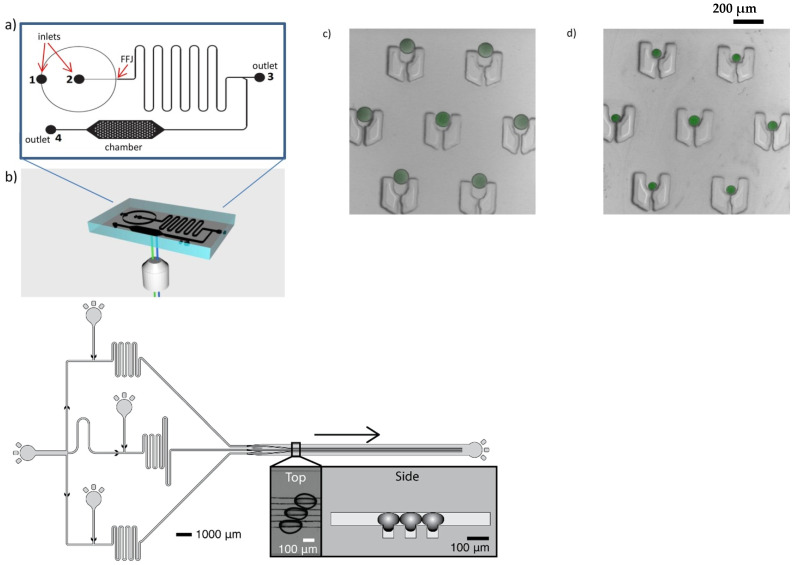
Top: Microfluidic device (**a**) and setup (**b**) for the analysis of triglyceride digestion and lipophilic micronutrient release from trapped droplets. Confocal fluorescence images of trapped tricaprylin droplets containing beta-carotene before (**c**) and after 24 min intestinal digestion (**d**). Reprinted from [134] with permission from Elsevier. Bottom: Microfluidic device for the analysis of bioactive compound transport kinetics between aqueous droplets, as designed in [137]. Image courtesy of Katherine Elvira, University of Victoria, Canada.

Further lab-on-a-chip droplet digestion works were conducted to investigate the kinetic interplay between triglyceride digestion and lipophilic micronutrient release (Figure 12) [134,135]. The release kinetics of beta-carotene or retinyl palmitate from individually trapped droplets differing in triglyceride composition was monitored on-chip using confocal fluorescence microscopy. For these systems, the release kinetics of these lipophilic compounds was found to be controlled by the intestinal digestion kinetics of the triglycerides. Gastric digestion enhanced subsequent intestinal digestion and lipophilic compound release for the saturated medium chain triglyceride (tricaprylin) and, to a lesser extent, for monounsaturated long chain triglycerides (high-oleic sunflower seed oil), but not at all for fish oil (mainly polyunsaturated long chain triglycerides). Degradation of beta-carotene due to oxidation could also be assessed during gastric tricaprylin digestion, accounting for about half of the total loss (the other half being due to release). Gastric degradation of beta-carotene due to oxidation was not significant for the other triglycerides, and was never significant in the intestinal digestion conditions. These experiments, together with buffer experiments, showed that the main cause of degradation was the low pH in gastric digestion conditions [134].

Besides these physicochemical processes related to nutrition, the biological process of cellular absorption was also studied on-chip, using droplet microfluidics. Phospholipid-stabilized aqueous droplets mimicking cells were formed in line and brought into contact in a microchannel, one in two containing fluorescein as a model bioactive compound. The transport kinetics of fluorescein was monitored directly in the microfluidic device using fluorescence microscopy. Similarly, the transport kinetics of caffeine was also monitored directly using micro-spectroscopy [136]. Recently, this approach was revisited to enable the formation of a line of three droplets, with different aqueous environments representing the intestinal lumen, the enterocyte cytoplasm, and the blood lumen (Figure 12). Transport kinetics of fluorescein from intestine to enterocyte to blood droplet compartments could be monitored directly in the microfluidic device using fluorescence microscopy [137].

## 5. Promising Developments for Food and Nutrition

### 5.1. Droplet Microfluidics for Biochemistry and Microbiology

A few years ago, droplet microfluidics started to be used as a high-throughput screening method for various omics purposes in the fields of biochemistry and molecular biology [138]. In the context of nutrition, cell biology especially benefited from droplet microfluidic developments. For example, an automated droplet microfluidic chip was designed to analyze adipose tissue physiology related to lipid storage and release. An on-chip lock-in fluorescence detection system was designed to detect either fatty acid uptake by adipose tissues [139], or glycerol release from adipose tissues to aqueous droplets [140], upon conditions representing the fed or the fasted state. This integrated droplet microfluidic platform allowed fast changes to be monitored with unprecedented temporal and molecular resolutions.

**Figure 13:**
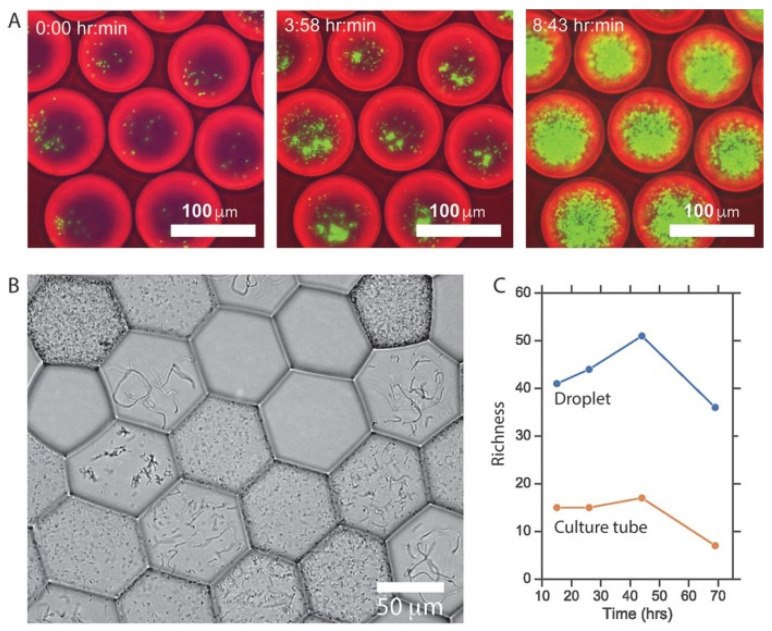
Top (**A**): *E. coli* growth in droplets. Bottom (**B**,**C**): Colony morphologies across droplets of an artificial community of five gut microbes and richness of microbial communities isolated and cultured in droplets compared with communities grown without separation in standard bulk culture. Reprinted from [141] under the terms of the Creative Commons Attribution 4.0 International license.

Other recent studies mostly focused on gut microbiota functions, particularly dietary compound degradation. For example, Villa et al. [141] investigated the growth and function on polysaccharides of dozens of microbial taxa encapsulated individually in microfluidic droplets (Figure 13). Approximately triple the amount of bacterial taxa could be cultivated, compared to usual batch culture methods. Human stool samples were found to differ mostly in their abundance of polysaccharide-degrading species, the most shared microbial taxa being able to degrade multiple polysaccharide types. A similar approach was taken to encapsulate sub-communities of a human stool microbiota, allowing their characterization at high genomic resolution. With this method, uncharacterized gut commensals were studied, and a novel member of the family *Neisseriaceae* was detected, and found to be involved in fatty acid degradation [142]. Another type of work was based on microfluidic individual droplet encapsulation of metagenomic clones in *E. coli* to screen human mucosal microbiota for an enzymatic activity involved in the degradation of human gangliosides and milk oligosaccharides. This approach allowed thousands of bacterial genomes to be covered in much less time and with much less substrate than conventional methods [143].

Although these applications are not yet directly related to the food and nutrition fields, it is obvious which promise they hold. In principle, not only do they allow us to bring processes related to physiology into the realm of food design for beneficial nutritional effects, but also to achieve this at unprecedented resolution and throughput.

### 5.2. Droplet Microfluidics-Analytical Instrument Coupling

Microfluidic tools are not only used to prepare samples for further analysis, but also used in close conjunction with analytical characterization. Microfluidic mixing coupled with confocal fluorescence microscopy was first used to resolve fast liquid mixing down to 10 µs [144]. Such setup was later used to study protein folding kinetics down to 8 µs mixing time [145]. More recently, droplet microfluidic setups, in combination with fluorescence or absorption spectroscopy detection, applied to enzyme kinetics studies were reviewed [146]. Most of the approaches presented in this review are transposable to food- and nutrition-related enzymes.

In a synchrotron setting, kinetic investigation via rapid mixing techniques, in combination with recent developments of small angle scattering, size-exclusion chromatography, or robotic sample changer are very useful to unravel the structure of biopolymers below the millisecond scale [147]. Synchrotron beamlines are approaching the current limits of mixing times around 100 µs [148]. Recent examples of mixing studies include folding of cytochrome c during rapid dilution with guanidine hydrochloride, and unfolding dynamics of ubiquitin upon exposure to guanidine hydrochloride in a multichannel microfluidic mixer [149,150].

High-throughput microfluidic methods were also developed for screening purposes. For example, an automated microfluidic platform for sample preparation and high-throughput structural SAXS investigation of proteins was recently designed, allowing the rapid screening of many solution conditions [151]. Similarly, in situ microfluidic dialysis for biological SAXS allowed the monitoring of structural changes in response to buffer exchange or protein concentration [152].

Optofluidic techniques, coupling optics, and microfluidics were recently identified as very promising for food and nutrition applications, including detection of food-borne pathogens, assessment of new food processing techniques, nutrition monitoring, effective health management of livestock, and optimized crop growth [153]. In optofluidic devices, droplets can be actively trapped, transported, and sorted using optically induced Marangoni effects. Moreover, various optofluidic methods are well-suited for detection and analysis in small droplets, making them an interesting alternative for food and drink testing. To this aim, fiber optic biosensors or surface plasmon resonance were used to detect multiple analytes in ultra-low volumes of sample directly on-the-chip, for instance to study the adulteration of foods such as extra virgin olive oil, beer, and milk.

Other coupled methods were devoted to the characterization of on-chip static or flowing materials, for example wide-angle and small angle X-ray scattering, Raman imaging, or dynamic light scattering. They allow the study of transitions, and building phase diagrams on-chip, which yet needs to be applied to food. Crystallization under finely controlled conditions within droplets of controlled size allows quantification of the nucleation probability and the growth kinetics, and measurement of the mutual diffusion coefficients of particle components during drying. A few food-related examples exist where such approaches were used: (i) to demonstrate the functionality of antifreeze protein to block ice crystal growth, or (ii) to evidence the potential of using controlled conditions of lipid emulsion crystallization for generating non-spherical fat particles.

## 6. Conclusions

This extensive review shows that droplet microfluidics is a mature field of science, suitable for the production of small structures relevant to food science and nutrition, such as monodisperse droplets and emulsions, or tailored biopolymer encapsulation systems. The fact that these structures can be made with unprecedented precision allows for highly precise food design that could also be tailored to generate health effects. Industrial applications already exist in the pharmaceutical and cosmetics sectors but, to date, high investment costs and low production throughputs make it difficult for droplet microfluidic technologies to enter food factories. The only application implemented so far is the detection of contaminants or pathogens in microfluidic devices for food safety control.

Application of microfluidic tools for analytical purposes is a field that is rapidly developing and is diverse in nature. For example, it allows for high speed/high resolution analysis of dynamic interfacial properties as well as lipid digestion kinetics, but also for charting phase behavior of food components, thus far mainly biopolymers. However, the level of development in other fields of science is such that it can be expected that high-throughput screening of many more food components for their chemical, physical, and technological properties is just around the corner.

In the future, it is expected that droplet-based approaches will be coupled with biological methods such as organs- or organoids-on-chip. Indeed, some links with biological and physiological processes are currently being explored (e.g., effects of protein structure on digestion, effects of gut microbiota on nutrient metabolism, etc.). The current trend of combining food and nutritional sciences to optimize food composition and structure in order to provide health benefits will surely be explored using droplet microfluidic and lab-on-chip devices integrating biological entities such as cells or microorganisms. In combination with high-precision analytical instruments, the microfluidic high control of sample volume, composition, structure, and environment will allow unknown properties, fast reaction kinetics, or specific interactions to be investigated, giving a broader understanding of the relations between food design and nutrition.

## Appendix A. List of Microfluidic Producer Websites

www.dolomite-microfluidics.com
www.elveflow.com
www.fluigent.com
www.micronit.com
www.ufluidix.com
